# Patient Perspective on Post-Breast Reconstruction Exercise and Physical Therapy

**DOI:** 10.1016/j.jpra.2021.09.002

**Published:** 2021-09-20

**Authors:** Margaret J. Higgins, Nisha Kale, Christopher Homsy, Kelsey L. Alabaster, Peter Ferrin, Cadence Miskimin, Abigail E. Chaffin, Mary K. Mulcahey

**Affiliations:** a Tulane University School of Medicine, 1430 Tulane Ave., New Orleans, LA, 70112; b Department of Orthopaedic Surgery, Tulane University School of Medicine, 1430 Tulane Ave., New Orleans, LA, 70112; c Division of Plastic and Reconstructive Surgery, Tulane University School of Medicine, 1430 Tulane Ave., New Orleans, LA, 70112; d Department of Surgery, Tulane University School of Medicine, 1430 Tulane Ave., New Orleans, LA, 70112

**Keywords:** Breast cancer, Breast reconstruction, Physical therapy

## Abstract

**Background:**

Breast reconstruction may result in significant functional compromise and pain. Postoperative exercise and physical therapy can mitigate these morbidities, but it is infrequently recommended by healthcare providers. This study asked how many breast reconstruction patients are instructed to perform postoperative at-home exercises or physical therapy, how many reported following through with these instructions, and what timeline they were given for these activities.

**Methods:**

A 16-question multiple-choice anonymous online survey was distributed to a private breast cancer survivor Facebook group (Diep C. Foundation).

**Results:**

A total of 150 breast reconstruction patients responded to our survey. The majority of respondents in our sample were not provided with specific instructions regarding postoperative at-home exercises (*N* = 70, 54.3%) or physical therapy (*N* = 77, 63.6%). Approximately 13 of 59 respondents (22%) who had been instructed to participate in postoperative at-home exercises were directed to begin at 2–3 weeks. Approximately 15 of 44 respondents (34.1%) who had been instructed to participate in physical therapy were directed to begin these at 4–5 weeks.

**Conclusions:**

To the best of our knowledge, this is the first study of how often postoperative at-home exercises and physical therapy are recommended to breast reconstruction patients. Despite robust evidence of these activities’ benefits, most women are not instructed to participate in postoperative at-home exercises or physical therapy. This is likely to impede breast reconstruction patients’ recovery and delay their return to activities of daily living. More studies are needed of how to actively engage breast reconstruction patients in postoperative at-home exercises and physical therapy.

## Introduction

Breast cancer is the second most common cancer diagnosis and the leading cause of cancer-associated death in women worldwide.^1^ The standard surgical treatment of nonmetastatic breast cancer consists of either a total mastectomy or an excision plus radiation, as well as sampling or removal of axillary lymph nodes.^2^ Although breast-conserving surgery as a primary therapy for early stage breast cancer is widely accepted as equivalent to mastectomy with regard to relapse-free and overall survival, recent data suggest that mastectomy rates are rising.^3^, ^4^, ^5^, ^6^

Mastectomy and invasive therapy to the axilla can lead to significant morbidity in the form of functional compromise and pain.^7^ Lymphedema, which is reported to occur in 6%–70% of breast cancer surgery patients depending on criteria for diagnosis and the follow-up interval, is a serious concern.^8^, ^9^, ^10^, ^11^ Decreased range of motion and impaired function of the affected shoulder joint, as well as decreased muscle strength, are additional complications that may occur as a result of lymphedema.^12^ When faced with these physical barriers, patients may need to alter their choice of clothing and daily activities including household duties, sleep, employment, and leisure time physical activity.^13^

Recent studies have focused on the benefits of postoperative physical activity in mitigating adverse side effects in mastectomy patients. McNeely et al. conducted a meta-analysis of 24 randomized clinical trials looking at the effect of exercise interventions on upper limb dysfunction secondary to breast cancer treatment.^14^ They concluded that the following variables may improve shoulder flexion and abduction range of motion and overall shoulder function: (1) Exercise implemented early in the postoperative period, considered to be days 1 through 3, and (2) structured exercise, including physical therapy.

Cho et al. conducted a study in breast cancer patients suffering from axillary web syndrome, a disorder causing visible or palpable cords of subcutaneous tissue in the breast, chest wall, or arm resulting from axillary node dissection.^15^ This disorder can lead to limited shoulder and elbow range of motion, resulting in pain and tightness. The researchers concluded that patients who participated in physical therapy experienced improvement in shoulder range of motion and muscular strength. These patients also showed improvement in emotional and social functioning and reduction in fatigue, believed to be a result of improved physical function.^15^

Despite evidence that exercise and rehabilitative programs can reduce the negative sequelae associated with mastectomy, these resources are rarely used. Cheville et al. conducted a survey of patients undergoing outpatient cancer treatment and found that 65.8% reported a functional problem related to their cancer treatment.^16^ Despite this, only two referrals for rehabilitation were generated for pain and limb swelling out of the 202 patients that participated in the study. Lack of time with patients, lack of exercise knowledge on the part of the health care provider, and concern for adverse side effects associated with exercise and physical therapy have all been identified as barriers to participation in cancer rehabilitation from the perspective of the clinician.^17^

To the best of our knowledge, no research exists to quantify the number of women referred for structured and/or unstructured exercise and rehabilitation programs following breast reconstruction. The purpose of this study was to determine how many breast reconstruction patients were instructed by their physicians to participate in post-operative exercise and physical therapy, how many patients reported following through with these instructions, and what timeline they were given to begin participation in these activities.

## Methods

### Survey Population

After obtaining approval from our Institutional Review Board, an anonymous 16-question online survey was posted via link to a private breast cancer survivor Facebook group (Diep C. Foundation) comprised of 4,600 women who had undergone breast reconstruction. The survey was created and distributed using Survey Monkey (SurveyMonkey Inc., San Mateo, California) and can be seen in Appendix 1. The survey was posted a total of three times by the administrator of the group at 2, 4, and 5 weeks after initial communication to encourage greater participation.

### Survey Content and Statistical Analysis

The first question asked whether the respondent had undergone breast reconstruction and if the patient answered yes, she was permitted to continue the survey. Six questions were related to the respondent's demographics. Eight questions examined at-home exercises following breast reconstruction and seven questions examined physical therapy following breast reconstruction. The remaining six questions examined physical activity level before and after breast reconstruction. Three questions were yes/no questions that prompted participants to answer additional questions depending on their response.

The survey data were downloaded from SurveyMonkey into SPSS Statistics version 21.0 (IMB, Armonk, New York) for analysis. All survey responses were categorized with anonymous identifiers. Categorical thresholds for ordinal and nominal data were analyzed by examining distributions of the raw data between respondents. Univariate analysis of categorical variables, including type of breast reconstruction and complications, was performed using the χ2 test. A value of *P* < 0.05 was considered statistically significant.

## Results

### Demographics of Respondents

The survey was distributed to women in a breast cancer survivor Facebook group, of which 150 total women responded. The majority of respondents were over the age of 44, with 54 respondents (54 of 136, 39.7%) reporting they were between the ages of 45–54, and 45 (45 of 136, 33.1%) were between the ages of 55–64. The majority of our sample had their breast reconstruction between the age of 45–54 (58 of 136, 42.6%) and 55–64 (43 of 136, 31.6%). Most respondents had their reconstructions performed in the South (68 of 128, 53.1%) or the West (24 of 128, 18.8%). The majority of women had a Deep Inferior Epigastric Artery Perforator (DIEP) Flap Reconstruction/Other Perforator Flap Reconstruction procedure performed (103 of 131 78.6%) (Table 1). Although many women (61 of 130 46.9%) did not report experiencing any complications from breast reconstruction, 23.1% (30 of 129) experienced fluid collection that required drainage, 23.1% (30 of 129) experienced wound breakdown at surgical site, 18.5% (24 of 129) required an additional surgery, and 15.4% (20 of 129) reported an infection (Table 2).

**Table 1:** Demographics of Respondents

Age (*N* = 136)	*N*	%
25-34	1	0.7
35-44	20	14.7
45-54	54	39.7
55-64	45	33.1
65-74	16	11.8

Age at Breast Reconstruction (*N* = 136)	*N*	%
25-34	3	2.2
35-44	24	17.6
45-54	58	42.6
55-64	43	31.6
65-74	8	5.9

Geographic Location (*N* = 128)	*N*	%
Northeast - New England (CT, ME, MA, NH, RI, VT)	7	5.5
Northeast - Middle Atlantic (NJ, NY, PA)	14	10.9
Midwest - East North Central (IN, IL, MI, OH, WI)	8	6.3
Midwest - West North Central (IA, KS, MN, MO, NE, ND, SD)	7	5.5
South - South Atlantic (DE, DC, FL, GA, MD, NC, SC, VA, WV)	30	23.4
South - East South Central (AL, KY, MS, TN)	1	0.8
South - West South Central (AR, LA, OK, TX)	37	28.9
West - Mountain (AZ, CO, ED, NM, MT, UT, NV, WY)	8	6.3
West - Pacific (AK, CA, HI, OR, WA)	16	12.5

Type of Breast Reconstruction (*N* = 131)	*N*	%
Implant reconstruction	16	12.2
DIEP flap reconstruction/Other Perforator flap reconstruction	103	78.6
TRAM flap reconstruction	2	1.5
Other	10	7.6

**Table 2:** Complication Types for Breast Reconstruction Patients

Complication Type (*N* = 177)	*N*	%
Fluid Collection at Surgical Site	30	23.10%
Wound Breakdown	30	23.10%
Infection	20	15.40%
Need for Additional Surgical Procedure	24	18.50%
None	61	46.90%
Other	12	9.20%

### At Home Post-Operative Exercises Following Breast Reconstruction

The majority of women in our sample (70 of 129, 54.3%) were not provided with specific instructions regarding recommended postoperative at-home exercises. Sixty of these women (60 of 70, 85.7%) wished that they had received this information. In addition, significantly more women who were not provided with specific instructions regarding postoperative at-home exercises reported experiencing complications following breast reconstruction compared to women who were provided instructions (37 of 69, 53.6%, *P =* 0.025), including a significantly higher incidence of wound breakdown at the surgical site (21 of 30, 70.0%, *P =* 0.04) and other complications (10 of 12, 83.3%, *P =* 0.026). 55.0% (11 of 20, *P =* 0.57) of women who were not provided instructions reported infection at the surgical site, and 54.2% (13 of 24, *P =* .99) reported need for additional surgeries.

When asked what sources of information these women used to learn about postoperative at-home exercises following their breast reconstruction, respondents stated they received information in the form of a handout from their doctor (49 of 133, 36.8%) or used other sources of information (33 of 133, 24.8%) including google searches, Facebook support groups, and YouTube.

**Figure 1:**
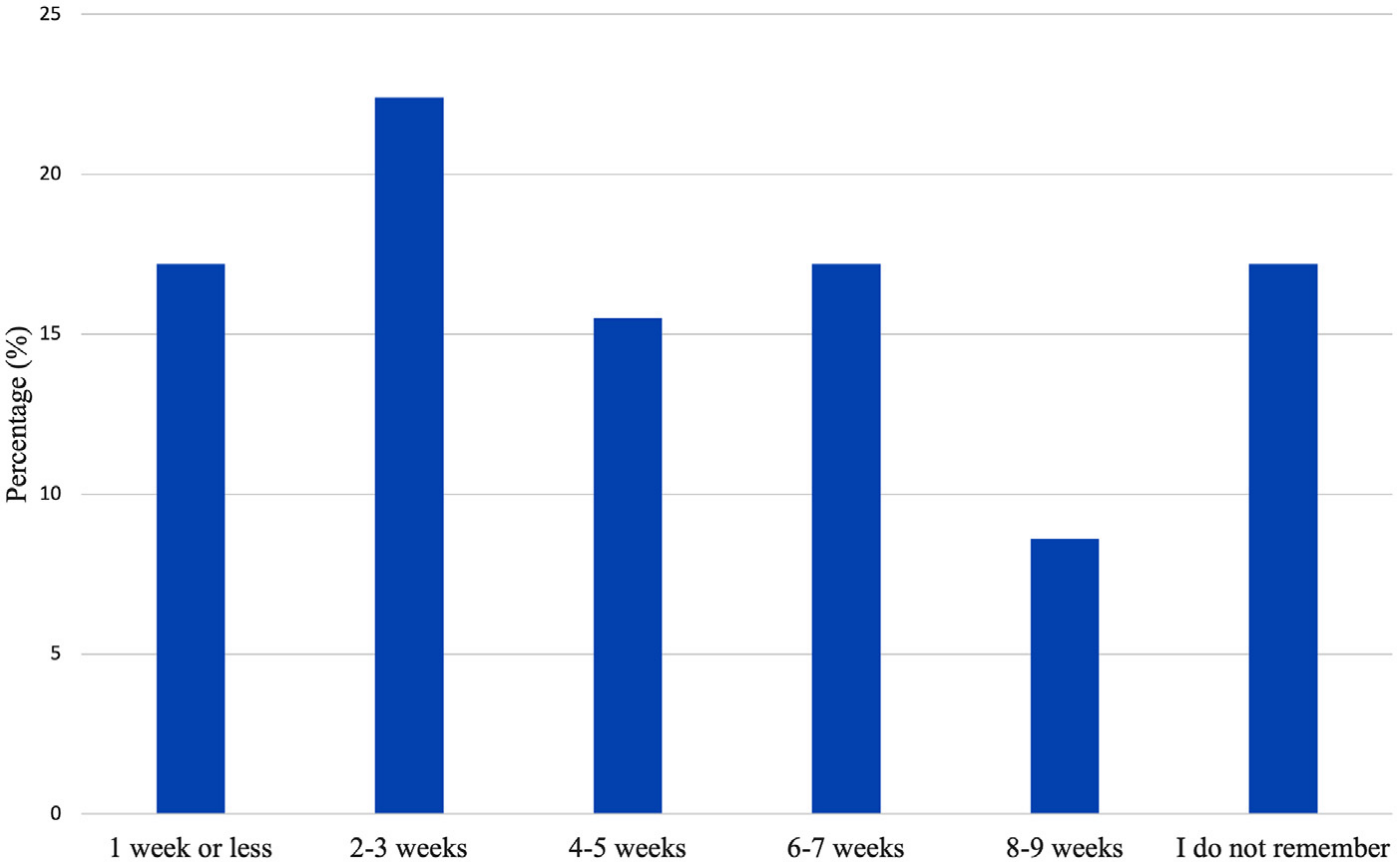
Instructions On When To Begin Post-Breast Reconstruction At-Home Exercises

Respondents that were provided with instructions to perform postoperative at-home (N = 59) exercises were instructed to begin doing so at varied time intervals (Figure 1). Most women who were assigned postoperative at-home exercises (55 of 58, 94.8%) reported performing them exactly as instructed, and 43.1% (25 of 58) stated that the exercises improved their post-operative pain. Compared to women who completed physical therapy (3 of 37, 7.9%), significantly more women who completed postoperative at-home exercises were unsure about the effect of the exercises on their postoperative pain (16 of 57, 28.1%, *P =* 0.005) (Table 3).

**Table 3:** Breast Reconstruction Patients Who Participated in Postoperative At-Home Exercises

When did you participate in postoperative at-home exercises?	*N*	%
1 week or less	10	17.2
2-3 weeks	13	22.4
4-5 weeks	9	15.5
6-7 weeks	10	17.2
8-9 weeks	5	8.6
I do not remember	10	17.2
Not applicable - I was never told to do at-home exercises after my breast reconstruction	1	1.7

Did you complete the postoperative at-home exercises as directed?	*N*	%
Yes	54	94.74%
No	3	5.26%

Did postoperative at-home exercises improve or worsen your postoperative pain?	*N*	%
Improved	*25*	43.86%
Worsened	2	3.51%
Unsure	16	28.07%
N/A	14	24.56%

**Figure 2:**
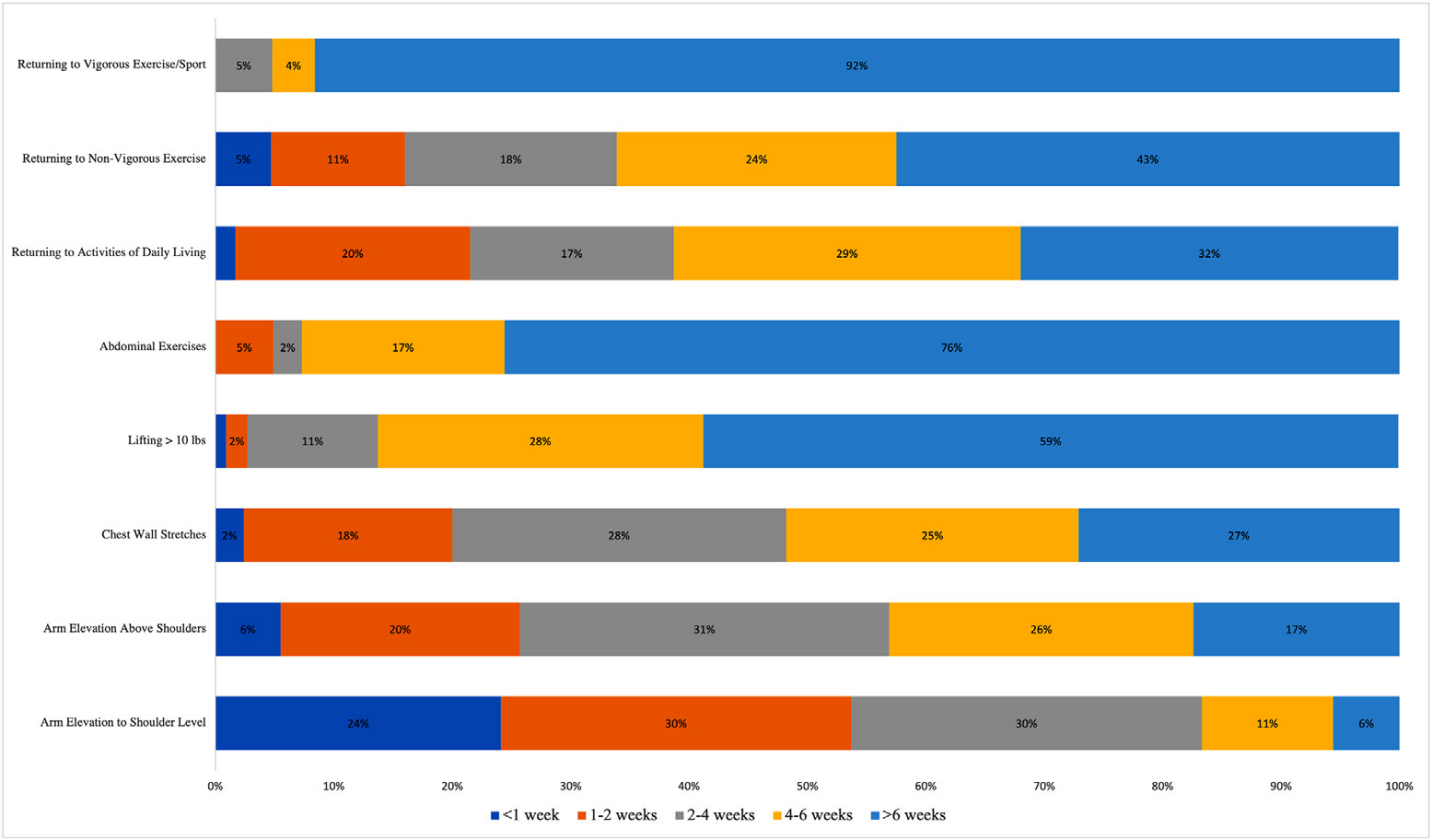
Types of At-Home Exercises Performed by Patients Following Breast Reconstruction

Post-operative exercises performed by respondents between <1 week, 1-2 weeks, 2-4 weeks, 4-6 weeks and >6 weeks are listed in Figure 2. Most respondents returned to activities of daily living 4 weeks following their breast reconstruction (71 of 116, 61.2%). Most women were counseled on specific postoperative activities to avoid (71 of 88, 80.7%), the majority of whom were instructed to avoid lifting their arms over their head (40 of 71, 56.3%) or participate in weight-bearing exercise (71 of 71, 100%). Women were also instructed to avoid strenuous activities like swimming or running (37 of 71, 52.1%), and several women were also instructed to avoid driving (7 of 71, 9.9%).

### Physical Therapy Following Breast Reconstruction

Most women in our sample were not prescribed or recommended to participate in physical therapy after breast reconstruction (77 of 121, 63.6%). 53.2% (41 of 77) of women who did experience complications were not prescribed or recommended physical therapy (*P =* 0.010). Most women who were not prescribed/recommended physical therapy wished they had the opportunity to participate (45 of 77, 58.4%). The majority of women prescribed physical therapy had it covered by their insurance (36 of 38, 94.7%).

Respondents who were prescribed/recommended physical therapy most frequently reported beginning therapy 4-5 weeks after their breast reconstruction (15 of 44, 34.1%) and completed their physical therapy as assigned (37 of 44, 84.1%). The majority found that physical therapy improved their post-operative pain (33 of 38, 86.8%). 40.8% (49 of 120) of women were not concerned or hesitant to participate in physical therapy following breast reconstruction, although many participants indicated that they were never encouraged to begin physical therapy following breast reconstruction (60 of 120, 50.0%) (Table 4). Women received information about physical therapy in the form of handouts from their doctor (49 of 120, 40.5%) and other sources (46 of 121, 38.0%) including individual internet searches (18 of 30, 60.0%) and specifically requesting referrals (10 of 30, 33.3%).

**Table 4:** Breast Reconstruction Patients Who Received Physical Therapy

When did you participate in physical therapy?	*N*	%
1 week or less	1	2.3
2-3 weeks	4	9.1
4-5 weeks	15	34.1
6-7 weeks	7	15.9
8-9 weeks	7	15.9
I do not remember	9	20.5
Not applicable	1	2.3

Did you complete the physical therapy as directed?	*N*	%
Yes	37	84.1
No	7	15.9

Was physical therapy covered by your health insurance?	*N*	%
Yes	36	94.7
No	2	5.3

Did physical therapy improve or worsen your postoperative pain?	*N*	%
Improved	33	89.19%
Worsened	1	2.70%
Unsure	3	8.11%

### Exercise and Activity Levels Following Breast Reconstruction

Women in our sample were very active and most reported exercising more than twice a week both pre-cancer diagnosis and pre-breast reconstruction (89 of 119, 74.8%). Most respondents were concerned or hesitant to return to exercising following breast reconstruction (70 of 119, 58.8%). The majority of women who were concerned or hesitant reported concern that exercise would disrupt wound healing (50 of 70, 71.4%), and others were worried it would increase pain (25 of 70, 35.7%). Women also wrote in responses stating that they were worried about “fatigue and lost strength” or “doing damage to my flaps/breast.” 50.3% (76 of 151) of women reported returning to pre-breast reconstruction exercise levels, 47.4% (36 of 76) reporting doing so within 2-3 months. Many women reported that they did not return to their previous exercise level (43 of 119, 36.1%).

## Discussion

This study suggests that many women are not being instructed to participate in postoperative at-home exercises or physical therapy following breast reconstruction. Women who are prescribed postoperative at-home exercises and physical therapy are likely to follow through with these instructions. Most women who did not receive instructions to participate in postoperative at-home exercises and physical therapy wished that they had received this information.

The absence of robust scientific evidence regarding the utility of physical therapy following breast reconstruction presents a major barrier to clinicians’ recommending these practices, as does delayed integration of physical therapy into routine cancer care.^7^ A study conducted by Silver et al. evaluated patient-related cancer rehabilitation content on National Cancer Institute-designated cancer center websites and found that 69% had no description of cancer rehabilitation services.^18^ The authors found that 21% of these websites required a search to find a description of cancer rehabilitation services and only 10% had an identifiable link.^18^

Logistical barriers faced by patients, as well as inconsistent adherence by patients to interventions, may also contribute to the underutilization of these resources. A study conducted by Gollhofer et al. evaluated breast cancer patients experiencing cancer-related fatigue during radiotherapy and their willingness to participate in a resistance exercise program.^19^ The authors found that patients needing longer travel time, those living alone, those with comorbidities, and those who had undergone chemotherapy were significantly more likely to decline participation. By far the most frequently reported reason for declining participation was the length of commuting time to the training facility.^19^ A related study by Mock et al. found that among breast cancer survivors, those that regularly exercised before their diagnosis were better able to maintain an exercise program during and after treatment.^20^ Although our results showed a high rate of compliance among patients prescribed postoperative at-home exercises and physical therapy, it was not 100%, suggesting that patient compliance remains an important variable to consider.

The point at which to begin postoperative at-home exercises and physical therapy following breast reconstruction requires careful consideration. Scaffidi et al. conducted a study comparing outcomes of patients who had undergone mastectomy, lumpectomy, or breast reconstruction and had participated in early rehabilitation in the form of daily physical therapy sessions starting on postoperative day one versus patients who received at-home rehabilitation instructions only. The authors concluded that patients in the intervention group showed significant betterment in shoulder-arm mobility as well as improved functionality, and fewer required a prescription for further physical therapy at 180-days follow up.^21^ Woo et al. found that late rehabilitation following breast reconstruction was a significant risk factor for sustained shoulder morbidity, suggesting that shoulder dysfunction following breast reconstruction can be successfully managed with early postoperative rehabilitation therapy.^22^ However, Shamley et al. conducted a meta-analysis on seroma formation in breast cancer surgery patients and found that delaying shoulder mobilization exercises significantly decreases the incidence of seroma formation.^23^ Patients who completed our survey reported substantial variation in when they had been instructed to begin their postoperative at-home exercises and physical therapy, suggesting that further study is required to determine which timeline would yield the most favorable outcomes.

A number of patients in our study experienced an improvement in post-operative pain upon completion of both at-home exercises and physical therapy. Persistent pain following breast cancer surgery has multiple pathogenic mechanisms including nerve damage and adjuvant treatment.^24^ Tasmuth et al. surveyed women one year following surgery for breast cancer and found that the incidence of chronic pain was 24% in the breast region and 17% in the ipsilateral arm.^25^ In addition to physical considerations, psychosocial factors may also play a role in the development of pain following breast cancer surgery. Katz et al. found that among patients who had breast cancer surgery, the risk of clinically meaningful acute postoperative pain was increased among women who were younger, unmarried, had more invasive surgeries, and had greater preoperative emotional distress.^26^

Physical therapy to address lymphedema can improve pain following breast cancer treatment. Hamner et al. concluded that patients who participated in decongestive physical therapy for lymphedema after breast cancer treatment not only experienced a reduction in lymphedema volume, but also reported a reduction in average pain on a numerical scale from 0 to 10 from 6.9 on their initial test to a 1.1 at eight weeks after starting treatment.^27^

There are several limitations to this study. First, distributing our survey to women in a Facebook group prevents us from determining how many women viewed our survey. Second, the results may not be generalizable to all women who have undergone mastectomy and reconstruction because this Facebook group may not be representative of this population. Third, many of the survey questions were subjective and could be interpreted variably. In particular, information regarding post-operative complications such as infection or wound breakdown may be biased because the definitions of these complications are variable. Additionally, there may be recall bias regarding the timing of beginning postoperative at-home exercises and physical therapy. This study did not collect information from respondents regarding the impact of postoperative at-home exercises and physical therapy on their shoulder range of motion or upper body function in activities of daily living. Our study did not address the potential that some patients may have been directed to deliberately delay initiation of post-operative exercises or physical therapy in order to prevent complications. Our survey did not inquire about how long patients were instructed to refrain from certain activities following their breast reconstruction. Finally, the women who chose to complete this survey may have been more motivated to participate in rehabilitation, leading to their willingness to participate in the survey.

## Conclusion

To our knowledge, this is the first study of how often postoperative at-home exercises and physical therapy are recommended to breast reconstruction patients. Despite robust evidence of these activities’ benefits, most women are not instructed to participate in postoperative at-home exercises or physical therapy. This is likely to impede breast reconstruction patients’ recovery and delay their return to activities of daily living. More studies are needed of how to actively engage breast reconstruction patients in postoperative at-home exercises and physical therapy.

## Declaration of Competing Interest

Dr. Mary Mulcahey is a board member for American Academy of Orthopaedic Surgeons, The Forum, American Journal of Sports Medicine Electronic Media, American Orthopaedic Society for Sports Medicine, Arthroscopy Association of North America, and Ruth Jackson Orthopaedic Society. Dr. Mary Mulcahey is also an editorial board member for Arthroscopy and Ortho Info. Dr. Mary Mulcahey is a paid presenter for Arthrex, Inc.

For the remaining authors, none were declared.

## Appendix Supplementary materials

Image, application 1
