# Supplementary material for: Patient Perspective on Post-Breast Reconstruction Exercise and Physical Therapy
Source: JPRAS Open. 2021 Sep 20;30:160–9. doi: 10.1016/j.jpra.2021.09.002 (PMC8529390; doi:10.1016/j.jpra.2021.09.002)
Supplement: Supplementary file 1 [file mmc1.docx]

**Patient Perspective on Post-Breast Reconstruction Exercise and Physical Therapy**

1. Have you undergone breast reconstruction following a mastectomy?
   1. Yes - with implants → Allowed to continue survey
   2. Yes - with my own tissue → Allowed to continue survey
   3. Yes – a combination implants and my own tissue 🡪 Allowed to continue survey
   4. No → Survey discontinued
2. What is your age?
   1. 18-24
   2. 25-34
   3. 35-44
   4. 45-54
   5. 55-64
   6. 65-74
   7. 75-84
   8. 85+
3. What was your age at the time of your breast reconstruction?
   1. 18-24
   2. 25-34
   3. 35-44
   4. 45-54
   5. 55-64
   6. 65-74
   7. 75-84
   8. 85+
4. In which geographic region did you have your breast reconstruction?
   1. Northeast - New England (CT, ME, MA, NH, RI, VT)
   2. Northeast - Middle Atlantic (NJ, NY, PA)
   3. Midwest - East North Central (IN, IL, MI, OH, WI, WV)
   4. Midwest - West North Central (IA, KS, MN, MO, NE, ND, SD)
   5. South - South Atlantic (DE, DC, FL, GA, MD, NC, SC, VA, WV)
   6. South - East South Central (AL, KY, MS, TN)
   7. South - West South Central (AR, LA, OK, TX)
   8. West - Mountain (AZ, CO, ED, NM, MT, UT, NV, WY)
   9. West - Pacific (AK, CA, HI, OR, WA)
5. What type of breast reconstruction did you have?
   1. Implant reconstruction
   2. DIEP flap reconstruction/Other Perforator flap reconstruction
   3. TRAM flap reconstruction
   4. Latissimus dorsi muscle flap reconstruction
   5. I am not sure
   6. Other - please specify
6. Did you experience any of the following after your breast reconstruction surgery? Check all that apply.
   1. Fluid collection at your surgical site that required drainage
   2. Wound breakdown at your surgical site
   3. Infection at your surgical site
   4. Need for another surgical procedure due to a complication
   5. Other - please specify
   6. None

**The following questions are regarding at-home post-operative exercises following your breast reconstruction.**

1. Were you provided with specific instructions regarding recommended at-home postoperative exercises after your breast reconstruction?
   1. Yes
   2. No
      1. If yes, directed to →  After what period of time were you told you could begin doing postoperative exercises at home?
         1. 1 week or less
         2. 2-3 weeks
         3. 4-5 weeks
         4. 6-7 weeks
         5. 8-9 weeks
         6. I do not remember
         7. Not applicable – I was never told to do home exercises after my surgery
      2. If yes, directed to → Did you perform the at-home postoperative exercises as directed?
         1. Yes – I did the exercises exactly as directed
         2. Yes – I did the exercises, but did them less frequently than directed
         3. No – I did not do the exercises
         4. Not applicable – I was never told to do home exercises after my surgery
            1. If yes, directed to 🡪 Did you find that **postoperative at-home exercises** improved or worsened your post-operative pain?

Improved my postoperative pain

Worsened my postoperative pain

Unsure the effect on my postoperative pain

No effect on my postoperative pain

Not applicable – I was never told to do home exercises after my surgery

- - 1. If no, directed to 🡪 If you did not receive specific instructions about doing **at-home postoperative exercises**, do you wish that you had received this information?
       1. Yes
       2. No

1. How soon after breast reconstruction did you do each of these exercises? Check all that apply. Please complete the chart to the best of your ability.
   1. I don’t recall when I did any of these activities.

|  | Arm elevation to shoulder level | Arm elevation above shoulders | Chest wall stretches | Lifting > 10 lbs | Abdominal exercises | Returning to Activities of Daily Living | Returning to non-vigorous exercise | Returning to vigorous exercise/ sports |
| --- | --- | --- | --- | --- | --- | --- | --- | --- |
| <1 week |  |  |  |  |  |  |  |  |
| 1-2 weeks |  |  |  |  |  |  |  |  |
| 2-4 weeks |  |  |  |  |  |  |  |  |
| 4-6 weeks |  |  |  |  |  |  |  |  |
| >6 weeks |  |  |  |  |  |  |  |  |

1. Were you counseled on specific postoperative exercises that you were to avoid? (Examples: running, swimming, weight lifting, raising your arms above a certain point, heavy lifting, etc.)
   1. Yes - please specify
   2. No
2. Did you use any of the following resources to find information about post-operative at home exercises following your breast reconstruction?
   1. Website that you were directed to by your doctor
   2. Handout from your doctor
   3. Other - please specify
   4. None

**The following questions are regarding physical therapy following your breast reconstruction.**

1. Were you prescribed/recommended to participate in physical therapy after your breast reconstruction?
   1. Yes
   2. No
      1. If yes, directed to → After what period of time were you told you could begin participating in physical therapy?
         1. 1 week or less
         2. 2-3 weeks
         3. 4-5 weeks
         4. 6-7 weeks
         5. 8-9 weeks
         6. I do not remember
         7. Not applicable – I was never encouraged to begin physical therapy after my breast reconstruction
      2. If yes, directed to → Did you complete/follow through with the physical therapy?
         1. Yes
         2. No
         3. Not applicable – I was never encouraged to begin physical therapy after my breast reconstruction
            1. If yes, directed to 🡪 Did you find that **physical therapy** improved or worsened your post-operative pain?

Improved

Worsened

Unsure

Not applicable – I did not do physical therapy after my breast reconstruction

- - - - 1. If yes, directed to 🡪 Was the **physical therapy** prescribed by your physician covered by your health insurance?

Yes

No

Unsure

Not applicable – I was never encouraged to begin physical therapy after my breast reconstruction

- - 1. If no, directed to 🡪 If you were not instructed to participate in **physical therapy** after your breast reconstruction, do you wish you had been?
       1. Yes
       2. No

1. Were you concerned or hesitant to participate in physical therapy following your breast reconstruction?
   1. Yes
   2. No
   3. Not applicable – I was never encouraged to begin physical therapy after my breast reconstruction
      1. If yes, directed to → Why were you worried about participating in physical therapy following your breast reconstruction? Please check all that apply.
         1. Worried that it would increase pain
         2. Worried that it would disrupt wound healing
         3. Not applicable – I was never encouraged to begin physical therapy after my breast reconstruction
         4. Not applicable – I was not worried about participating in physical therapy
         5. Other - please specify

**The following questions are regarding exercise/physical activity following your breast reconstruction.**

1. Were you concerned or hesitant to return to exercise following your breast reconstruction?
   1. Yes
   2. No
      1. If yes, directed to → Why were you worried about returning to **exercise** following your breast reconstruction? Please check all that apply.
         1. Worried that it would increase pain
         2. Worried that it would disrupt wound healing
         3. Not applicable – I was not concerned about returning to exercise after my breast reconstruction
         4. Other - please specify
2. What was your pre-cancer diagnosis activity level?
   1. Daily exercise
   2. Exercise 4+ times per week
   3. Exercise 2-3 times per week
   4. Exercise 1 or fewer times per week
   5. No exercise
3. What was your pre-breast reconstruction activity level?
   1. Daily exercise
   2. Exercise 4+ times per week
   3. Exercise 2-3 times per week
   4. Exercise 1 or fewer times per week
   5. No exercise
4. Did you return to your pre-breast reconstruction activity level?
   1. Yes
   2. No
      1. If yes, directed to → How long did it take you to return to your pre-breast reconstruction activity level?
         1. 1 week
         2. 2 weeks
         3. 3 weeks
         4. 1 month
         5. 2-3 months
         6. Longer than 3 months
         7. Not applicable – I was not able to return to my pre-breast reconstruction activity level
